# Point-of-care testing for measuring haemolymph glucose in invertebrates is not a valid method

**DOI:** 10.1093/conphys/coz079

**Published:** 2019-11-28

**Authors:** Silas C Principe, Alessandra Augusto, Tânia M Costa

**Affiliations:** 1 São Paulo State University (UNESP), Biosciences Institute, Botucatu Campus, R. Prof. Dr. Antônio Celso, 250, 18618-000, Botucatu, São Paulo, Brazil; 2 São Paulo State University (UNESP), Biosciences Institute, Coastal Campus, Praça Infante Dom Henrique, s/n, P.O. Box: 73601, 11380-972, São Vicente, São Paulo, Brazil; 3 São Paulo State University (UNESP), CAUNESP, Prof. Paulo Donato Castellane, s/n, 14884-900, Jaboticabal, São Paulo, Brazil

**Keywords:** fiddler crab, glucose, *Leptuca thayeri*, molluscs, *Perna perna*, portable meter

## Abstract

Blood glucose is widely used as a physiological parameter for vertebrates and invertebrates. However, its measurement in the field is often difficult due to the need for expensive and non-portable equipment. Point-of-care (POC) devices, originally intended for human use, are increasingly being used for measuring blood parameters of animals in the field. In this regard, POC glucose meters are becoming valuable tools for conservation physiologists, as glucose can be a useful indicator of stress response. In invertebrates, the use of POC glucose meters is still scarce, and no study yet has evaluated their usability in crustaceans and molluscs. We tested if a POC device can be used to measure haemolymph glucose in two widely used models, *Leptuca thayeri* and *Perna perna*, compared with a standard laboratory method. The device was unable to measure glucose in *P. perna* haemolymph due to equipment inaccuracy and low glucose concentration in this species (10.13 ± 6.25 mg/dL). Additionally, despite the device being capable of measuring glucose in *L. thayeri* haemolymph, Bland–Altman plots showed a strong bias and wide limits of agreement, and Lin's concordance correlation coefficient showed a weak concordance between methods. When simulating experimental conditions, POC results differed from those found using the standard method. We conclude that POC glucose meters are unsuitable for assessing glucose in mussels and should not be used in crabs as results are inaccurate.

## Teaser

Lay summary

Measuring physiological parameters in the field is a challenge, so point-of-care devices are increasingly being used by conservation physiologists to assess blood parameters in non-human animals. We tested a glucose meter in two invertebrates (crab and mussel) and highlight the need for caution when using such devices in invertebrates.

## Introduction

Glucose is a valuable blood parameter in physiology studies, being widely used to evaluate response to stressors in both vertebrates (e.g. fishes, Jentoft *et al*., 2005; birds, Remage-Healey and Romero, 2000; reptiles, Valverde *et al.*, 1999) and invertebrates (e.g. crabs, Lu *et al.*, 2015; Rajendiran *et al.*, 2016; mussels, Hannan *et al.*, 2016). Factors that cause stress lead to alteration or loss of homeostasis in animals, affecting their fitness and reproductive capacity (e.g. Petes *et al.*, 2007). In these cases, mechanisms involved in the control of homeostasis use energy stored in different tissues to reestablish equilibrium (Bayne, 1973; Webster, 1996; Lu *et al.*, 2015).

Glucose (derived mainly from the conversion of glycogen) is the main energy provider in crustaceans (Full and Herreid II, 1984) and molluscs (de Zwaan and Zandee, 1972), and physiological mechanisms that spend Adenosine Triphosphate (ATP) are dependent upon this type of carbohydrate. Thus, an increase in glucose concentration is common during stressful situations. Because of that, glucose has long been used as a physiological parameter in studies with crustaceans (e.g. Briffa and Elwood, 2001, 2002; Matsumasa and Murai, 2005). Also, aquaculture researchers are including glucose as a parameter in studies dealing with the quality of farmed crustaceans (Hall and van Ham, 1998; Lu *et al.*, 2015, 2016). In crabs (Decapoda), changes in glucose concentration are related to circadian rhythm (Matsumasa and Murai, 2005), exposure to air (Santos and Keller, 1993a; Webster, 1996; Lu *et al.*, 2016), pollutants (Reddy *et al.*, 1996), aggression (Briffa and Elwood, 2001, 2002; Aquiloni *et al.*, 2012) and salinity or temperature increase (Chang *et al.*, 1998; Lu *et al.*, 2015). In molluscs, glucose has been used as a stress parameter in studies with bivalves (Wells *et al.*, 1998; Martínez-Pita *et al.*, 2012), limpets (Shore *et al.*, 1975) and sea hares (Carefoot, 1991).

During stressful conditions, hormones and other substances may be secreted to stimulate release of glucose stored in the tissues. In crustaceans, levels of glucose in the haemolymph are controlled by the crustacean hyperglycemic hormone (CHH) (Webster, 1996; Chung *et al.*, 2010), which can induce hyperglycemia during stress (Hall and van Ham, 1998). CHH is a neuropeptide produced mainly by the X-organ/sinus gland complex of the eyestalks that promotes the mobilization of glycogen stored in the haepatopancreas (Santos and Keller, 1993b; Fanjul-Moles, 2006). This occurs through activation of phosphorylases and inhibition of glycogen synthase (Sedlmeier, 1985; Webster, 1996). Other substances may be involved with the increase in circulating glucose, such as epinephrine (Model *et al.*, 2019) and melatonin (Yang *et al.*, 2018) in crustaceans and epinephrine (Massarsky *et al.*, 2011) and insulin-like peptides (Pertseva *et al.*, 2013) in molluscs.

The most common method for glucose measurement is the colorimetric assay based on the enzymatic oxidation of glucose (Trinder, 1969). It is widely used by physiologists dealing with both vertebrates (e.g. Kavadias *et al.*, 2003) and invertebrates (e.g. Vinagre *et al.*, 2007). Although a well-established method, its use in the field is impractical as it demands several equipment and materials that are heavy, delicate and expensive (Stoot *et al.*, 2014). Recently, there has been an increase in the use of point-of-care (POC) devices, primarily designed for human health, on animal physiology (Brown *et al.*, 2008; Awruch *et al.*, 2011; Andrewartha *et al.*, 2016; Bennett *et al.*, 2017). POC devices are portable, easy to use and generally cheaper than well-established lab techniques. Also, they provide a fast and reliable way to assess blood parameters in the field (Stoot *et al.*, 2014; Lindholm and Altimiras, 2016). Among POC devices, glucose meters are one of the most used in animal physiology studies (Stoot *et al.*, 2014). The ability of these devices to assess glucose relies on the oxidation of glucose by glucose oxidase or the conversion of glucose into gluconolactone by glucose 1-dehydrogenase (Rebel *et al.*, 2012).

The majority of studies employing POC testing have so far focused on vertebrates, especially fishes (e.g. Beecham *et al.*, 2006; Braz-Mota *et al.*, 2015; Ball and Weber, 2017; for a complete and recent review, see Stoot *et al.*, 2014). In crustaceans, Butcher *et al.* (2012) were capable of measuring haemolymph glucose in the giant mud crab *Scylla serrata* using the Accutrend Plus (Roche Diagnostics, Australia) meter, while Aliko *et al.* (2015) measured glucose levels in the green crab *Carcinus aestuarii* using the OneTouch Ultra (Lifescan, Johnson & Johnson) meter. To the best of our knowledge, only two other studies employed POC testing in crustaceans: Lorenzon *et al.* (2008) measured haemolymph glucose to evaluate stress in *Cancer pagurus* subjected to different transport systems, and Lorenzon *et al.* (2004) evaluated the effect of dopamine, serotonin and L-enkephalin on haemolymph glucose in the stomatopod *Squilla mantis* and the decapod *Astacus leptodactylus*. In both cases, authors used the OneTouch II (Lifescan, Johnson & Johnson) meter. However, none of those studies intended to validate the use of glucose meters with the studied animal, and none compared results with a standard lab technique. Two studies (Matsumasa and Murai, 2005; Matsumasa *et al.*, 2013) successfully used glucose meters to evaluate haemolymph glucose in fiddler crabs, but those particular devices are not considered portable as they demand additional batteries, and they are originally intended for laboratory use. In molluscs, the only study that used a POC device evaluated the glycogen content of mussel seeds, but not the levels of free haemolymph glucose (Sim-Smith and Jeffs, 2011). As for other invertebrates, POC testing has also been used in the horseshoe crab *Limulus polyphemus* (Xiphosura) (Allender *et al.*, 2010).

When using a novel method in substitution to a well-established one, it is important to first evaluate how well the new method performs against the standard one and also validate its use, assessing the need for previous calibration (Lindholm and Altimiras, 2016). Although many studies tested the applicability of POC testing in vertebrates (e.g. Lieske *et al.*, 2002; Beecham *et al.*, 2006; Ball and Weber, 2017; Bennett *et al.*, 2017), none have so far assessed its relevance to invertebrates. Nevertheless, studies are being conducted in invertebrates using POC glucose meters without previous tests (e.g. Lorenzon *et al.*, 2004; Lorenzon *et al.*, 2008; Allender *et al.*, 2010; Butcher *et al.*, 2012). Thus, the aim of this study was to evaluate the applicability of a personal glucose meter to assess glucose levels in the haemolymph of one crustacean species and one mollusc species. This was done using a widely available and inexpensive device that can be easily bought and that has already been used in other studies with vertebrates. We compared the results of the glucose meter with a well-established lab technique and advise that it should not be used in substitution to the standard method for either of the tested animals.

## Materials and methods

### Animal models

To evaluate whether POC glucose meters can be used in crustaceans and molluscs, we selected two species widely used as models: *Leptuca thayeri*, a fiddler crab, and *Perna perna*, a Mytilidae mussel. *Leptuca thayeri* are common and abundant in mangroves on the east Atlantic coast, inhabiting muddy and shaded areas (Gusmao-Junior *et al.*, 2012; Thurman *et al.*, 2013). As other fiddler crabs, they play a crucial role in the cycling of nutrients in the mangrove, acting as ecosystem engineers (Kristensen and Alongi, 2006; Kristensen *et al.*, 2008). Because of their importance, they have been used in studies in ecology (Cuellar-Gempeler and Munguia, 2013; De Grande *et al.*, 2018b; De Grande *et al.*, 2018c), animal behaviour (Gusmao-Junior *et al.*, 2012; De Grande *et al.*, 2018a) and physiology (Thurman *et al.*, 2010; Principe *et al.*, 2018). *Perna perna* are a common mussel on east Atlantic rocky shores, forming large patches in the intertidal region (Marques *et al.*, 1991; Hicks *et al.*, 2001; Henriques and Casarini, 2009). In addition to their ecological importance, they are also part of human diet (Marques *et al.*, 1991). Studies with *P. perna* include those in ecotoxicology (Santana *et al.*, 2018), physiology (Nogueira *et al.*, 2017; Rola *et al.*, 2017) and ecology (McQuaid and Lawrie, 2005; Lathlean and McQuaid, 2017).

### Animal collection and sampling

*Leptuca thayeri* were collected in the Portinho mangrove, Praia Grande, São Paulo, Brazil (23°59' S; 46°24' W), during low tide in January and July 2018. Crabs were sampled by burrow excavation, and *L. thayeri* intermolt adult males with carapace width (CW) between 16 and 24 mm (Farias *et al.*, 2014), measured with a calliper, were used for the assessment of glucose levels. We used 31 crabs for the methods comparison (mean, CW 18.3 ± 1.37 mm) and 30 crabs for the validation experiment (mean, CW 19.5 ± 1.5 mm).

For the methods comparison, immediately after collection, crabs were cleaned with distilled water and dried with absorbent paper. Individuals were anaesthetized on ice for 10 minutes. A haemolymph sample was withdrawn from the ventral haemocoel using a 1-ml tuberculin syringe through a puncture on the fifth pereiopod. Samples were put into 0.5-ml microtubes and stored on ice until fieldwork was finished. After that, samples were frozen at –16°C prior to use.

*Perna perna* were collected with scrapers from a rocky shore in Praia dos Sonhos, Itanhaém, São Paulo, Brazil (24°11' S, 46°48' W), during low tide in July 2018. After collection, organisms were measured using a calliper. Only adult individuals with shell length between 31 and 45 mm were used (McQuaid *et al.*, 2000). We used 30 individuals for the methods comparison (mean, shell length 36.9 ± 3.7 mm). Once measured, organisms were anaesthetized on ice, and a haemolymph sample was withdrawn from the base of the abductor muscle using a 1-ml tuberculin syringe. Samples were put into 0.5-ml microtubes, stored on ice until fieldwork was finished, and then frozen at –16°C prior to use.

### Methods comparison

For this comparison, measurements of both methods were carried out in the laboratory concurrently. Despite our aim of testing a device that could be easily used in the field, this procedure was done to ensure that samples were subjected to the same handling conditions. The same method was used for both *L. thayeri* and *P. perna*. Samples were thawed out and then used for assessment of glucose by the two different methods. First, a commercial kit (Glicose Liquiform Ref. 133, LabTest Diagnóstica, Brazil) was used to measure haemolymph glucose concentration using the colorimetric method (Trinder, 1969). This method is a standard procedure for the assessment of glucose concentration (Briffa and Elwood, 2001, 2002; Kavadias *et al.*, 2003; Vinagre *et al.*, 2007) and was set as the laboratory control method. Following this procedure, 10 μl of haemolymph were put into a 1.5-ml tube. After that, each tube received 1 ml of the commercial reagent and was incubated for 10 minutes at 37°C. Samples were read in a spectrophotometer in the 505-nm band. Another 1 μl of haemolymph was used to read glucose via the glucose meter Accu-Chek Performa (F. Hoffmann–La Roche AG). In the review by Stoot *et al*. (2014), Accu-Chek was considered the most used glucose meter (different models considered), capable of reading glucose levels ranging from 10 to 600 mg/dL. The device’s ability to assess blood glucose relies on the electrochemical method: test strips contain a mutant variant of quinoprotein glucose dehydrogenase (Mut. Q-GDH), which converts glucose into gluconolactone, producing a measurable electrical current (Accu-Chek Performa User Guide, F. Hoffmann–La Roche AG). Accuracy of the device depends on glucose concentration, but for concentrations lower than 100 mg/dL, 81.5% of samples are within ±5 mg/dL of error, according to the manufacturer. Samples were put directly onto a disposable test strip (from the same manufacturer) and were inserted into the glucose meter, following manufacturer instructions. Each reading lasted <5 seconds.

### Validation of experimental conditions

This experiment was only performed with *L. thayeri* as comparison tests showed that the POC device was unable to read *P. perna* haemolymph glucose. To test if the glucose meter could be used in experimental conditions, we performed an experiment with two treatments, (i) control (25°C) and (ii) high temperature (35°C). Crustacean glucose levels can increase due to thermal stress (Lu *et al.*, 2016), so we expected that animals exposed to high temperatures would show higher levels of haemolymph glucose. We used 30 animals for this experiment, 15 for each treatment. Prior to experimentation, animals were acclimated for 48 hours in aquariums with brackish water (salinity of 25, corresponding to high tide salinity in the area) with constant aeration and ambient temperature (~25°C). Individuals were not covered by water so they still had access to the air. No more than 10 individuals were kept in each aquarium.

After acclimation, animals were individually put in plastic flasks (500 ml) with brackish water (salinity of 25). Individuals were not covered by water and had access to the air. The control group was exposed to a temperature of 25°C, and crabs in the high temperature treatment were exposed to 35°C. This temperature can be easily reached in the areas where *L. thayeri* occur and was used in a previous study with this species (Principe *et al.*, 2018). Flasks were placed into a water bath with the corresponding temperature for each treatment, and organisms were exposed for 24 hours to the experimental conditions. At the end of the experiment, animals were dried with absorbent paper, and a sample of haemolymph was collected following the same procedures described previously. Haemolymph glucose level was assessed in the lab using the laboratory method and POC device, following the comparison experiment. Where the POC glucose meter was unable to read the sample due to it being below the minimum detected value (10 mg/dL), the measurement was replaced by half of the lowest value as done by Butcher *et al.* (2012).

### Statistical analyses

For the methods comparison, a linear regression was used to assess the relationship between values of glucose concentration obtained with POC testing and the laboratory method. However, as stated by Lindholm and Altimiras (2016), linear regressions alone are not enough to elucidate the reliability of a new method against an established one. Thus, we used the Bland–Altman method (Altman and Bland, 1983) to evaluate the discrepancy between methods. This same approach was used in other works that assessed agreement between methods, including POC devices (e.g. Awruch *et al.*, 2011; Burdick *et al.*, 2012). Here, a plot is constructed with the differences between measurements (i.e. standard method and POC device) against measurement averages. The analysis also returns the bias of the new method (mean difference between measurements) and the limits of agreement, calculated as the mean ± 1.96 × SD, which includes 95% of the differences between measurements. Following the original work of Altman and Bland (1983), mean difference between measurements (and the resulting bias) is shown as the laboratory method (standard) minus POC device (new method). Thus, a negative bias would mean an overestimation of the new method compared with the standard one. To assess whether the difference between methods was sensitive to differences in the concentration of haemolymph glucose (i.e. showing data heteroscedasticity), we applied a Pearson correlation between the absolute difference and the average of values obtained through the different methods (Atkinson and Nevill, 1998; Lindholm and Altimiras, 2016). Also, we used Lin’s concordance correlation coefficient (CCC) (Lin, 1989) to evaluate agreement between methods (Lindholm and Altimiras, 2016).

To validate results for the experimental condition, we used an estimation approach and constructed a Cumming estimation plot. Estimation methods offer advantages over the common null-hypothesis approach by enabling researchers to estimate effects sizes and their associated uncertainty (Ho *et al.*, 2019). In the Cumming estimation plot, the raw data are plotted on the upper left axes, and a bar associated with each group shows the mean and standard deviation of the data. Below the raw data, mean differences between groups (which is the effect size) and their 95% confidence interval (CI) are plotted. The 95% CI is derived through a non-parametric bootstrap resampling, bias corrected and accelerated. The plot was produced using the DABEST package for R (Ho *et al.*, 2019). As we understand that the null hypothesis is still the most used approach, we also performed a Student’s t-test (independent samples) comparing glucose values. This was done for the results obtained with the two methods (i.e. one test for each method), so we could assess if both methods were capable of showing the same results (presence or absence of statistical difference). Except when indicated, data are shown as mean ± SD. All statistical analyses were performed using R 3.5.3 (R Core Team, 2019).

## Results

### Methods comparison

**Figure 1:**
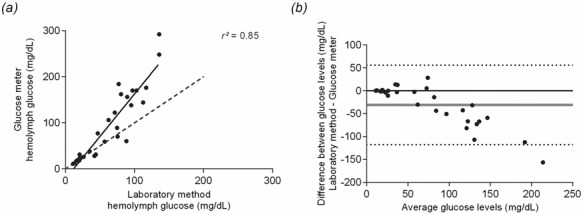
Comparison between methods of glucose measurement (**a**) Linear regression of fiddler crab haemolymph glucose measured using a POC device and via the laboratory method. The grey dotted line is the expected line of perfect agreement, and the solid line is the estimated slope. (**b**) Bland–Altman plot of glucose measurements via a POC device and the laboratory method. Solid grey line represents bias, and dotted lines represent limits of agreement (95%).

Glucose levels in fiddler crabs were successfully measured using a POC device in 29 out of 31 crabs. In two cases, glucose concentration was below the limit of measurement (i.e. 10 mg/dL), and these were discarded in the subsequent analysis. Levels of haemolymph glucose measured by the laboratory method ranged from 11.11 mg/dL to 135.71 mg/dL (mean, 62.23 ± 38.70 mg/dL). Levels of glucose measured by the glucose meter ranged from 11 to 292 mg/dL (mean, 93.10 ± 77.33 mg/dL). Linear regression results (Fig. 1a) showed a positive relation between glucose meter results and the laboratory method (r^2^ = 0.85, F_1,27_ = 152.3, *P* < 0.0001).

**Figure 2:**
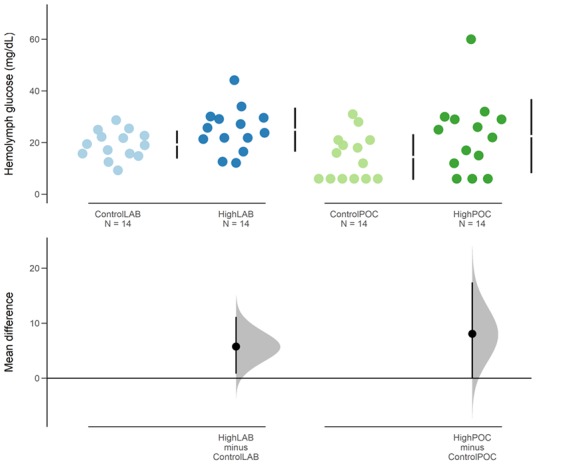
Glucose levels of *L. thayeri* exposed to control and high temperature conditions. Samples were read using two different methods (laboratory method (LAB) and POC device), and comparisons between treatments for each method are shown in the above Cumming estimation plot. Glucose values are plotted on the upper axes with vertical lines indicating mean (as a gap) ± SD. Each mean difference is plotted on the lower axes as a bootstrap sampling distribution. Mean differences are depicted as dots; 95% CIs are indicated by the ends of the vertical error bars. Significant difference was found just for glucose levels obtained via the laboratory method (*P* < 0.05).

However, Bland–Altman results showed that POC measurements have a marked deviation from the colorimetric method, depicted by a large bias (–30.88 ± 44.29 mg/dL) with wide limits of agreement (–117.7 and 55.94; Fig. 1b). The glucose meter generally estimated higher values of haemolymph glucose, at ~30.88 mg/dL, compared with the laboratory method. Limits of agreement indicate that glucose concentrations obtained through the device are expected to be placed between 55.94 mg/dL below and 117.7 mg/dL above the values obtained via the laboratory method in 95% of measurements. Also, Pearson correlation between absolute differences and the average of glucose levels obtained through the methods show that there is heteroscedasticity in the data (*r* = 0.92; *P* < 0.001). That means concentration of glucose in the haemolymph affects the degree of deviation from the standard method, with deviation increasing in higher concentrations of glucose in the haemolymph. Additionally, Lin’s CCC showed a poor concordance between methods (PC = 0.65; 95% CI = 0.51, 0.75). This indicates results obtained with the POC device deviate from the standard method, and this device is not appropriate for obtaining accurate levels of fiddler crab haemolymph glucose.

*Perna perna* glucose levels obtained via the laboratory method ranged between 1.94 and 20.39 mg/dL (mean glucose concentration, 10.13 ± 6.25 mg/dL). All measurements performed using the glucose meter were below the device’s limit of measurement (LO error, corresponding to low values). Even in cases in which values obtained using the standard laboratory method were higher than the lower limit of the device, POC testing could not provide a value. In that way, the glucose meter was unusable to assess glucose in *P. perna*.

### Validation of experimental conditions

We successfully measured glucose levels using the POC device in 9 out of 15 animals in the control group and in 11 out of 15 animals in the high temperature treatment. One outlier in each treatment was excluded from the subsequent analysis following Wilkinson (1996). Mean glucose levels were 19.24 ± 5.42 mg/dL (control) and 25 ± 8.51 mg/dL (high temperature). Estimation plots revealed distinct patterns for data obtained using the two methods (Fig. 2). The mean difference (i.e. the effect size) of glucose levels between treatments obtained through the laboratory method was 5.75 mg/dL (95% CI = 0.83–11.1), while for the results obtained through the POC device, the mean difference was 8.07 (95% CI = 0.07–17.4). Despite the effect size for temperature increase obtained using the laboratory method being lower than the one obtained via the POC device, CIs of the effect size were wider for the POC method, as the standard deviations of the data in this method.

We also performed a Student’s t-test between treatments for each method. There was a significant difference between glucose levels using the laboratory method in animals exposed to control conditions and the high temperature treatment [t(26) = –2.13, *P* < 0.05]. However, difference between treatments was not found when comparing glucose levels assessed via the POC device [t(26) = –1.79, *P* > 0.05]. Mean glucose levels obtained using the glucose meter were 14.43 ± 8.82 (control) and 22.50 ± 14.33 (high temperature).

## Discussion

POC devices have been used to assess glucose levels in a variety of animals, especially vertebrates (Stoot *et al.*, 2014). Validation assays have been done in many groups (e.g. fish, Beecham *et al.*, 2006; Ball and Weber, 2017; mammals, Bennett *et al.*, 2017; birds, Lieske *et al.*, 2002), but none have evaluated the real applicability of such technology in invertebrates. From the works that have used glucose meters in crustaceans (Lorenzon *et al.*, 2004, 2008; Butcher *et al.*, 2012; Aliko *et al.*, 2015), none have tested their usability or validated the method prior to use. Here we showed that although the POC device was capable of measuring glucose levels in the haemolymph of a fiddler crab, results differed from those obtained with the laboratory method, with poor concordance between methods. Also, we found that for mussels, the glucose meter was incapable of reading haemolymph glucose, probably due to the low levels of glucose in *P. perna* haemolymph. These results demand caution when using POC devices in invertebrates.

Primarily designed for patients in home care, POC testing provides an accurate and fast way to obtain diagnostics that can drive clinical decisions (Price, 2001). The most widespread POC application is to monitor blood glucose, helping diabetic patients control their insulin levels (Klonoff, 2005; Rebel *et al.*, 2012). Having been designed for human care, the use of these devices in other taxa demands validation procedures (Stoot *et al.*, 2014; Lindholm and Altimiras, 2016). For a variety of wild animals, POC devices are indeed capable of producing accurate results: an example is found in a Salmonidae fish by Iwama *et al.* (1995), where the POC device produced accurate results comparable with the lab method (for other cases, see Stoot *et al.*, 2014). However, their functionality in some taxa may be limited because physiological differences from humans, especially on blood composition, may produce inaccurate results. For example, POC glucose meters provided inaccurate results for the white-tailed deer (*Odocoileus virginianus*), possibly from low concentration of blood glucose or high haematocrit concentration (Burdick *et al.*, 2012). For the bonefish *Albula vulpes*, results deviated in a way that they should be used as reference values only (Cooke *et al.*, 2008). Given the difference between invertebrate and vertebrate blood and physiology, measurements taken via POC testing should differ from the standard method as found in our study.

When measurements from a new method deviate from the standard one, results may still be applicable to comparative studies, when the exact values are unimportant but variation between treatments or collection sites is still relevant. For example, in Beecham *et al.* (2006), authors evaluated the usability of a POC glucose meter for assessing glucose in catfish. In that case, values of glucose measured by the meter were lower than those obtained in the lab. However, these were able to show statistical differences between animals subjected to different stress treatments. Cooke *et al.* (2008) were capable of using a glucose meter to assess the effects of different capture techniques on the physiological conditions of the bonefish *A. vulpes*, despite concluding that POC device values should be used just as relative values. In our study, glucose values of *L. thayeri* found via POC testing were not enough to show the same statistical results found with the laboratory method. Thus, the applicability of the POC glucose meter for comparative studies is not possible.

**Table 1:** Haemolymph glucose levels of some marine and freshwater Crustacea and Mollusca representatives.

	Species	Glucose (mg/dL)	Citation
Crustacea			
	*L. thayeri*	19.24 ± 5.42	This work (validation experiment)
	*L. thayeri*	28.89 ± 17.69	Principe *et al.*, 2018
	*Minuca rapax*	27.8 ± 15.62	Principe *et al.*, 2018
	*Callinectes sapidus*	7.9 ± 1.3	Chung, 2014
	*Chasmagnathus granulata*	6.8 ± 0.9	Vinagre and Da Silva, 2002
	*Eriocheir sinensis*	89.73 ± 4.68	Long *et al.*, 2018
	*Leptuca beebei*	30 ± n.a.	Matsumasa *et al.*, 2013
	*O. quadrata*	8.11 ± 0.72–15.31 ± 5.04^a, b^	Vinagre *et al.*, 2007
	*Portunus pelagicus*	37.3 ± 0.52	Saravanan *et al.*, 2018
	*Scylla paramamosain*	14.5 ± n.a.	Liu *et al.*, 2019
	*S. serrata*	6.41 ± 0.62	Reddy and Kishori, 2001
			
Mollusca			
	*P. perna*	10.13 ± 6.25^b^	This work (field samples)
	*Elliptio crassidens*	2.1 ± 0.30	Fritts *et al.*, 2015
	*M. edulis*	4.14 ± 0.90	Sheir and Handy, 2010
	*Villosa vibex*	2.0 ± 0.19	Fritts *et al.*, 2015

Values are either mean or range (a) and correspond to animals in experimental control conditions or field data (b). n.a. means that standard deviation was not available. For more details about methods, please refer to the cited work.

Our results, however, do not seek to entirely exclude usage of glucose meters in crustaceans or molluscs. For example, vertebrates from the same group may show different results in validation assays (e.g. Salmonidae, Iwama *et al.*, 1995; Wells and Pankhurst, 1999; for examples in other taxa, see the review by Stoot *et al.*, 2014). However, our results do highlight the need for validation before using POC devices in invertebrates. Several reasons may lead to the failure of POC glucose meters in providing accurate results when used with invertebrates. Glucose meters are calibrated for human blood, and thus differences in blood composition of other taxa lead to different values of glucose (for example, with birds; Lieske *et al.*, 2002). Assessing the difference between crab and mussel haemolymph and human blood is beyond the scope of this work, but investigating it may provide a starting point to develop suitable meters for those groups.

Lower glucose concentrations can also affect glucose meter results (e.g. Burdick *et al.*, 2012). Glucose levels in the haemolymph of invertebrates may be several times lower than in human blood, which is around 100 mg/dL (Danaei *et al.*, 2011). Table 1 shows the mean levels of glucose in the haemolymph of some freshwater and marine crustaceans and molluscs. Fiddler crab glucose levels are usually around 30 mg/dL, depending on physiological status and species (Matsumasa and Murai, 2005; Matsumasa *et al.*, 2013; Principe *et al.*, 2018), more than three times lower than human levels. Other species of crabs have similar levels of glucose, like the ghost crab *Ocypode quadrata* (Vinagre *et al.*, 2007) and the swimming crab *Portunus trituberculatus* (Lu *et al.*, 2015). Glucose levels in mussels can be even lower than those found in crabs. In controlled conditions, *Mytilus galloprovincialis*, a mytilid mussel, display glucose levels ~5 mg/dL (Martínez-Pita *et al.*, 2012; Faggio *et al.*, 2016); for *Mytilus edulis*, levels are ~10 mg/dL (Amachree *et al.*, 2014). For now, POC testing is unsuitable for *L. thayeri* and *P. perna*, and the same can be true for related species with similar ranges of glucose and haemolymph composition, such as other fiddler crabs and Mytilidae mussels.

Nevertheless, we do encourage researchers to test glucose meters in other species and groups of invertebrates. The potential applications of these devices in animal physiology studies are many, as field measurements of glucose are often difficult to perform. The only examples in fiddler crabs are the works from Matsumasa *et al.* (2013) and Matsumasa and Murai (2005), but these involved the adaptation of lab equipment for field use. For the majority of studies, usually, a sample is collected and then transferred to the laboratory for analysis via bench methods. However, this approach has the disadvantage of not providing immediate values, which can drive decisions in the field. Also, transport and maintenance of blood samples can be difficult and may cause sample degradation, leading to an increase in glucose levels, which can be minimized by field measurements (Clark *et al.*, 2011). Obtaining accurate glucose levels in crustaceans or molluscs in the field can indicate organism state in a particular situation such as a stressful condition or seasonal variation (Vinagre *et al.*, 2007; Buckup *et al.*, 2008). These results can be aggregated to laboratory experiments and enhance the prediction of environmental change effects. As fiddler crab and mussel species are expected to be harmed by climate change (Smith *et al.*, 2006; Levinton *et al*., 2015; Principe *et al*., 2018), such improvement in predictions can subsidize conservation efforts for those groups. Aquaculture professionals can also benefit from the use of POC devices for evaluating glucose of invertebrates during transport and in farms as these data can inform them about animal stress and welfare (e.g. Lu *et al*., 2016).

Glucose meters also have the advantage of being cheaper than bench methods. Accu-Chek Performa, the device used in this study, is sold in Brazil for ~US$16, and strips have the approximate price of US$0.5 per unit. Other models can be even more accessible. For comparison, the colorimetric kit used here costs US$0.08 per test but demands expensive equipment such as a spectrophotometer, which can cost >US$1500. In addition, measurement by glucose meters is simple and fast, and can substantially improve fieldwork efficiency, or the routine of aquaculture professionals.

POC devices serve as a reliable tool for field biologists working with a wide variety of animals, mainly due to their low cost and high feasibility (Stoot *et al.*, 2014). However, our results suggest that their use in invertebrates may be impractical. For the two animal models tested here, *L. thayeri* and *P. perna*, POC testing of haemolymph glucose is not applicable, and the same can be true for related species with similar ranges of glucose levels and haemolymph characteristics. We advise that, despite correction of device values through statistical methods being possible in some cases (having been used before for some groups, e.g. fish, Iwama *et al.*, 1995; birds, Harter *et al.*, 2015), this should not be used in cases similar to those found here, with wide limits of agreement and poor concordance. By doing this, one may risk generating deeply inaccurate results. We highlight the importance of tests done prior to adoption of a new method, especially for devices designed for human use.
